# Value of Magnetic Resonance Images and Magnetic Resonance Spectroscopy in Diagnosis of Brain Tumors under Fuzzy C-Means Algorithm

**DOI:** 10.1155/2022/3315121

**Published:** 2022-05-30

**Authors:** Huaiqin Liu, Qi Zhang, Shujun Niu, Hao Liu

**Affiliations:** Department of Radiology, Zibo Central Hospital, Zibo 255000, Shandong, China

## Abstract

This study was aimed to explore the diagnostic value of magnetic resonance imaging (MRI) and magnetic resonance spectroscopy (MRS) in brain tumors under the fuzzy C-means (FCM) algorithm. The two-dimensional FCM hybrid algorithm was improved to be three-dimensional. The MRI images and MRS spectra of 127 patients with brain tumors (low-grade glioma group) and 54 healthy people (healthy group) were analyzed. The results suggested that the membership matrix of the improved algorithm had lower ambiguity, higher segmentation accuracy, closer relationship of intrapixels, and stronger irrelevance of interclass pixels. Through the analysis of gray matter volume, it was found that, compared with the healthy group, the gray matter and white matter volumes in the brain of high-grade glioma were higher, and those of low-grade glioma group were lower. The improved FCM algorithm could obtain a higher accuracy of 88.64% in segmenting images. It had a higher sensitivity to gray matter changes in brain tumors, reaching 92.72%; its specificity was not much different from that of traditional FCM, which were 83.61% and 88.06%, respectively. In the diagnostic value, the area under the curve of mean kurtosis was the largest, which was 0.962 (*P* < 0.001). The best critical value was 0.4096, which had a greater reference significance for clinical treatment and prognosis. The ratio of choline/N-acetyl-aspartate and the ratio of choline/creatine also showed significant differences in high- and low-grade gliomas (*P* < 0.05), but the specificity and sensitivity were slightly lower. It also had guiding significance for the grading of gliomas. Overall, the improved FCM algorithm had obvious advantages in the segmentation process of MRI images, which provided help for the clinical diagnosis of brain tumors.

## 1. Introduction

Brain tumors are made up of primary and secondary ones. Among various types of intracranial tumors, gliomas occupy the first place in the morbidity, which is about 40%–45% [1].Glioma originates from human neuroepithelium and commonly includes astrocytoma, astroblastoma, and glioblastoma multiforme. Glioma is usually caused by the interaction of congenital genetic high-risk factors and environmental carcinogenic factors [2]. Due to its space-occupyingeffect, patients will have symptoms such as headache, nausea and vomiting, epilepsy, and blurred vision. Different pathological types of gliomas can also cause different clinical symptoms. For example, patients with optic glioma will lose vision to some extent, while patients with spinal glioma have symptoms such as limb pain, numbness, and muscle weakness [3, 4]. The degree of malignancy also affects the speed at which symptoms occur. Patients with low-grade gliomas often have a medical history of months or even years, while patients with high-grade gliomas usually have the history of weeks to months [5]. As the tumor continues to develop, the difficulty of surgical resection increases. Thus, early detection and diagnosis of glioma is very important to prevent the further development of the disease.

Medical imaging technology can be applied to detect abnormal changes in tissues of the body, and it becomes a necessary means for the determination of the treatment plan for brain tumors. There are many medical imaging technologies nowadays, including computed tomography (CT), magnetic resonance imaging (MRI), functional magnetic resonance imaging (FMRI), positron emission tomography (PET), and diffusion tensor imaging (DTI) [6]. Due to the diverse types of brain tumors, the development of imaging technologies, and the limitations of three-dimensional data, manual segmentation of brain tumor images showed the long time-consumption, inexperience, and poor repeatability. To deal with the brain tumor image segmentation effectively, many scholars have come up with some segmentation algorithms [7], but so far, the segmentation of brain tumor images has not been maturely applied clinically [8]. Magnetic resonance spectroscopy (MRS) is the only examination technology for noninvasive detection of chemical components in the body using the principle of chemical shift. Diffusion kurtosis imaging (DKI) is a new MRI technology on the basis of DTI technology, and it can reflect the water molecular diffusion in non-Gaussian distribution in biological tissues [9]. It has been found that the complexity of biological tissue structure is positively correlated with the DKI parameter value [10].

The different membership functions were adopted to set the target under fuzzy clustering algorithm here, and then the connection point of the functions was determined as the threshold by optimizing the target function [11]. The image segmentation process can also be regarded as a clustering process, and the advantage of fuzzy clustering is to utilize different membership functions to classify data points into multiple clusters. The commonly used clustering methods include fuzzy *C*-means (FCM) clustering, *K*-means algorithm, and expectation maximization algorithm [12]. It was to study MRI images of brain tumor and MRS spectra on the basis of FCM algorithm in this work. The two-dimensional hybrid of FCM algorithm was optimized to the three-dimensionalone, and the spectra were expanded to the distribution in the two-dimensional or even three-dimensional space. The improved algorithm was applied to the cerebral MRI images of 127 brain tumor patients in the low-grade glioma group as well as 54 healthy volunteers in the healthy group. This study was intended to provide a valuable reference for the clinical diagnosis of brain tumors.

## 2. Materials and Methods

### 2.1. Principles of FCM Algorithm

FCM algorithm is a kind of clustering algorithm, which is improved from the hard clustering. The core idea of the clustering algorithm was to find the appropriate membership and clustering center. That is, when the variance and iteration error of the clustering cost function were minimized, as a result, the value of the cost function was the weighted cumulative summation of the 2-norm measure from the pixel to the clustering center. It is assumed that *M*={*m*_1_, *m*_2_,…, *m*_*n*_} is the grayscale or eigenvalue of the image pixels, and *f* is the number of clusters (number of clustering centers) that divide *M*. The clustering centers are expressed as *A*={*a*_1_, *a*_2_,…, *a*_*f*_}, *b*={*b*_*xy*_} denotes the membership matrix, and *b*_*xe*_ means *m*_*x*_ belongs to the membership degree of the *e*-th class area. The cost function of FCM is expressed as

(1) $$\begin{array}{c} \min E_{l}B,A=\sum_{x=1}^{F}\sum_{y=1}^{n}b_{xy}^{l}r_{xy}^{2}. \end{array}$$

This equation satisfied the following equations:

(2) $$\begin{array}{c} \sum_{x=1}^{f}b_{xy}=1,\;1\leq y\leq n, \end{array}$$

(3) $$\begin{array}{c} \sum_{y=1}^{f}b_{xy}>0,\;1\leq x\leq f, \end{array}$$

(4) $$\begin{array}{c} b_{xy}=1\leq x\leq f,\;1\leq e\leq n. \end{array}$$

In the equations, *B* *=* *b*_*xy*_ is an *n *×* x* fuzzy membership matrix, which represents the size of the membership value of the *y*-th sample *m*_*y*_ belonging to the *x*-th class, with a range of 0–1. *l* is the weighted index, and *A*={*a*_1_, *a*_2_, ..., *a*_*f*_} is the *h *×* f* matrix composed of *f* clustering center vectors. *r*_*xy*_=‖*m*_*y*_ − *a*_*x*_‖ means the Euclidean distance from the sample point *m*_*y*_ to the clustering center *a*_*x*_, which is just the 2-norm measure from the pixel *m*_*y*_ to the clustering center.

For the minimization of the cost function *E* (*B*, *A*), the Lagrange multiplier method was used to construct the objective optimization function. The partial derivative of the clustering center *a*_*x*_ and the membership degree *b*_*xy*_ of the objective function was obtained, and the derivative result was set to zero. Then, the iterative update expressions for the clustering center and membership degree were worked out as

(5) $$\begin{array}{c} a_{x}={\left(\sum_{y=1}^{f}b_{xy}^{l}\right)}^{-1}\sum_{y=1}^{f}{\left(b_{xy}\right)}^{l}m_{y},x=1,2,...,f, \end{array}$$

(6) $$\begin{array}{c} T_{y}=\left\{\left(x,y\right)|m_{y}=a_{x},1\leq x\leq f\right\}. \end{array}$$

If *T*_*y*_=*β*, then the following equation can be obtained.

(7) $$\begin{array}{c} b_{xy}=\frac{1}{\left[\sum_{g=1}^{f}r_{gy}{}^{-1}r_{xy}\right]},x=1,2,\ldots ,f,\;y=1,2,\ldots ,n, \end{array}$$

If *T*_*y*_ ≠ 0, then *b*_*xy*_ is any nonnegative real number satisfying

(8) $$\begin{array}{c} \sum_{x=1}^{f}b_{xe}=1,\;b_{xe}\in \left[0,1\right]. \end{array}$$

The iterative equation for membership degree is a mapping from points to sets. In the actual calculation process, the following membership update equations are usually used.

(9) $$\begin{array}{c} b_{xy}={\left[\sum_{x=1}^{f}r_{gy}{}^{-1}r_{xy}^{2/l-1}\right]}^{-1},T_{y}\neq \beta \end{array}$$

(10) $$\begin{array}{c} b_{xy}={\left|T_{y}\right|}^{-1},T_{y}\neq \beta ,\;x\in T_{y}, \end{array}$$

(11) $$\begin{array}{c} b_{xy}=0,\;T_{y}\neq \beta ,\;x\notin T_{y}. \end{array}$$

In the equations, *I* represents the number of iterations of the function. It is assumed that the iteration equations (3) and (7) met the iteration termination conditions, that is, the iteration ended when *i* *>* *I* or *max*_*x*_=*a*_*x*_^*i*+1^ − *a*_*x*_^*i*^ < *α*. After the iteration ended, the pixels were classified according to the principle of the maximum membership degree. If *b*_*yx*_ > *b*_*ye*_, *m*_*y*_ is classified into the region of *x*-th class of clustering centers, where *e*=1,2, ..., *f*;  *x* ≠ *e*.

### 2.2. Improvement of FCM Algorithm

The FCM algorithm usually used Euclidean distance for clustering. The Euclidean distance metric function was suitable for clustering whose distribution was in spherical or ellipsoid shape. But when the clustering distribution did not belong to a specific shape, the Euclidean distance ignored the relationship among sample dimension features. It was inappropriate to use Euclidean distance in this case, so the kernel function was introduced to measure the distance between pixels in the space. The low-dimensional space was mapped to the high-dimensional space, and the complex nonlinear issue was transformed into the linear issue of kernel space [13, 14], enhancing the noise immunity of the algorithm. The steps of improving the FCM algorithm were given as follows.

Firstly, the images were intuitively blurred.

The original image was converted from the spatial domain to the fuzzy domain, and grayscale processing was performed on each pixel. For a grayscale image with a size of *C *×* D*, the grayscale level was in the interval [*m*_min_, *m*_max_]. The image was represented by an intuitionistic fuzzy set as

(12) $$\begin{array}{c} Q=\left\{m_{xe},b\left(m_{xe}\right),a\left(m_{xe}\right),\pi \left(m_{xe}\right)\right\},\;0<x\leq C,\;0<e\leq D. \end{array}$$

In the equation, *b*(*m*_*xe*_) is the membership degree of *m*_*xe*_, and *m*_*xe*_ is the gray level of the pixel (*x, e*), which described the degree of brightness of the grayscale value of the pixel. The membership and nonmembership were expressed as equations (13) and (14), respectively.

(13) $$\begin{array}{c} b\left(m_{xe}\right)={\left(b\left(m_{xe}\right)\right)}^{2}, \end{array}$$

(14) $$\begin{array}{c} a\left(m_{xe}\right)={\left(1-b\left(m_{xe}\right)\right)}^{2}. \end{array}$$

The hesitation of the image after intuition fuzzification is expressed as

(15) $$\begin{array}{c} \pi \left(m_{xe}\right)=2b\left(m_{xe}\right)\left(1-b\left(m_{xe}\right)\right). \end{array}$$

The grayscale value of each pixel could be computed through

(16) $$\begin{array}{c} m_{e}=\;\left(b\left(m_{xe}\right),a\left(m_{xe}\right),\pi \left(m_{xe}\right)\right). \end{array}$$

Secondly, the initialization parameters were given.

The membership matrix was extended to the grayscale range, and the intuitive fuzzification was performed on the grayscales of the image. The initial membership matrix *M* was then obtained. The number of clustering categories *f*, the spatial constraint parameter *θ*, the weighted index *l*, *δ* in the kernel function, and the neighborhood radius *p*, stopping threshold *α* of the iteration, and the maximum iteration number *I* were all set. The initial iteration number was set to be 0.

Thirdly, the local information of the pixel was calculated according to its principles.

Fourthly, the clustering center *A*^*∗*(*I*)^=*a*_*x*_^*∗*(*I*)^ was updated according to equations (3) and (4):

(17) $$\begin{array}{c} a_{x}^{*\left(I\right)}=\frac{\sum_{y=1}^{n}{\left(b_{xy}^{*\left(I\right)}\right)}^{l}\left(m_{y}+\theta {\bar{m}}_{y}\right)}{\sum_{y=1}^{n}\left(1+\theta \right){\left(b_{xy}^{*\left(I\right)}\right)}^{l}}. \end{array}$$

Fifthly, the membership function matrix *B*^|*I*+1|^=*b*_*xy*_^|*I*+1|^ was updated according to equation (5).

(18) $$\begin{array}{c} b_{xy}^{\left|I+1\right|}={\sum_{I=1}^{f}\left[\left(\frac{{\left\|\varphi \left(m_{y}\right)-\varphi \left(a_{x}\right)\right\|}^{2}+\theta {\left\|\varphi \left(m_{y}\right)-\varphi \left({\bar{a}}_{x}\right)\right\|}^{2}}{{\left\|\varphi \left(m_{y}\right)-\varphi \left(a_{p}\right)\right\|}^{2}+\theta {\left\|\varphi \left(m_{y}\right)-\varphi \left({\bar{a}}_{p}\right)\right\|}^{2}}\right)\right]}^{-1/l-1} \end{array}$$

Sixthly, the generated hesitation *π*_*xy*_^(*I*+1)^ was applied to modify the membership function matrix *B*^(*I*+1)^=*b*_*xy*_^(*I*+1)^, and then the following equations were obtained.

(19) $$\begin{array}{c} b_{ey}^{\left(I+1\right)}=\max\left\{b_{xy}^{\left(I+1\right)}\right\}, \end{array}$$

(20) $$\begin{array}{c} \left\{\begin{array}{c} b_{ey}^{\left(I+1\right)}=1-\pi_{xy}^{\left(I+1\right)}\underset{y\neq e}{\sum }b_{xy}^{\left(I+1\right)}\\ b_{xy}^{\left(I+1\right)}=\pi_{xy}^{\left(I+1\right)}b_{xy}^{\left(I+1\right)} \end{array}\right). \end{array}$$

Seventhly, it was judged whether the condition for iteration stopping, *B*^(*I*+1)^ − *B*^*I*^ < *α*, was satisfied. If the condition was met, the iteration ended. Otherwise, the fifth step was repeated for the next iteration in the case of *i* = *i *+* *1.

Eighthly, defuzzification of the image was done. The membership degree of the corresponding the grayscale level of the obtained intuitionistic fuzzy partition matrix was substituted into the image, and the classification of pixels was made according to the principles of maximum membership degree.

### 2.3. Verification of Segmentation Performance of the Algorithm

It was not objective enough to judge whether the improved algorithm was successful only by manual judgment. It was more convincing to judge the improved algorithm from the segmentation effect images and, on the other hand, from the quantitative analysis by introducing some evaluation indexes. For the comparison of the segmentation performance of the algorithm, three evaluation indexes—partition coefficient (Vpc), partition entropy (Vpe), and Xie-Beni index (Vxb)—were introduced to analyze the segmentation performance of the improved algorithm.

### 2.4. MRI Data

Common formats of MRI image data mainly include DICOM, Analyze, and NIFTI. 127 patients with brain tumor (low-grade glioma group) and 54 healthy people (healthy group) were included as the objects. All raw collected data were in the standard DICOM common format. Since the collected data was in DICOM format, it was necessary to process the collected data before extraction of the brain tissues. In this process, the MRIconvert (http://www.nitrc.org/projects/mricron) software was used to process the 164-layer images of the same object. The image was converted from a two-dimensional space in DICOM format to a three-dimensional space image in NITH format. 3T-MRI instrument scanning equipment was used, and the T1 structural images of the heads of all objects were obtained. The specific imaging parameters are shown in Table 1.

MRI had three imaging modes of *θ*1-weighted, *θ*2-weighted, and proton density-weighted images. After the values of *θ*1, *θ*2, *θa*, and *θb* were given, the image pixels of human tissues are

(21) $$\begin{array}{c} S\left(\theta 1,\theta 2,\theta a,\theta b\right)=\underset{n}{\sum }\beta_{n}\left(1-e^{-\frac{\theta a}{\theta 1}n}\right)e^{-\frac{\theta b}{\theta 2}} \end{array}$$

### 2.5. Preprocessing Procedure

**Figure 1:**
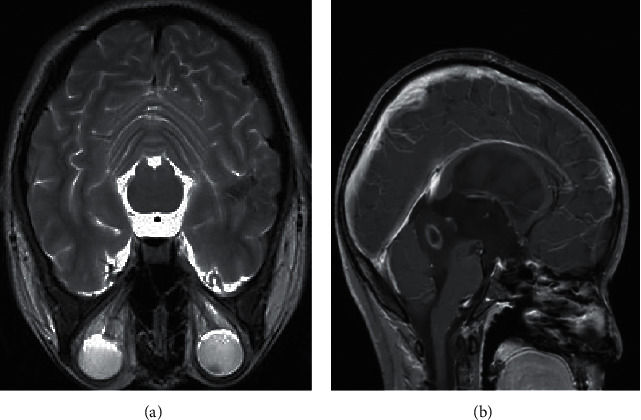
MRI image of brain structure. Image (a) is in the coronal plane, and (b) is in the sagittal plane.

Before segmentation, registration, analysis, and visualization of brain MRI images, the images must be preprocessed, including head movement correction, edge detection, and morphological optimization. The brain tissues in the image were extracted, but some nonbrain tissue parts such as scalp, muscles, and skull would have a certain impact on the segmentation results, resulting in mis-segmentation. Moreover, in the researches of diseased tissue in some brain areas, the brain was usually used as the research objects. If brain tissue image was to be segmented, the scalp, skull, and other nontissue components must be removed first, which would greatly reduce the effect of nonbrain tissue components on the segmentation [15, 16]. Then, the brain tissue image was further divided into gray matter and white matter, and the obtained results were more conducive to subsequent quantitative analysis. For the same type of image data, the demand angles were different, and the advantages and disadvantages of the segmentation methods were also different. In the process of brain MRI image segmentation, it was very important to remove nontissue components in the segmentation. The border-based segmentation method could effectively remove the interference of other information, and the method was simple and fast. The raw brain MRI images could be observed as shown in Figure 1.

**Figure 2:**
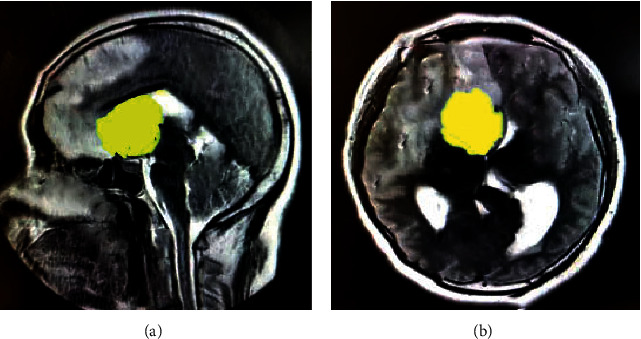
MRI images of brain tumor, where (a) is in the sagittal plane and (b) is in the coronal plane. The yellow part in the images indicated the tumor region.

Figure 2 shows the brain MRI images of a male patient with a clinical diagnosis of low-grade glioma. The tumor region could be clearly displayed by MRI technology.

**Figure 3:**
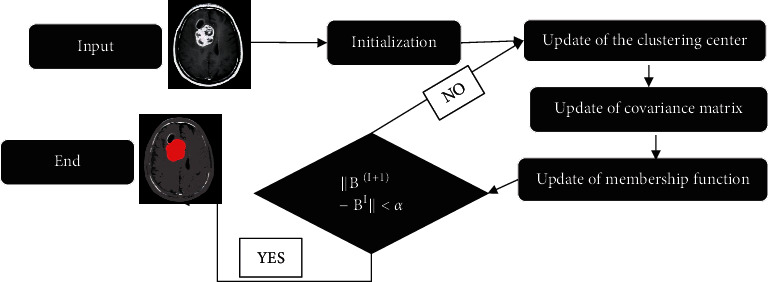
Segmentation flow chart.

For the experimental environment, CPU was Intel i7, and the memory was 8 g. The operating system adopted Windows 10, and the programming environment adopted MATLAB 2015b. All MRI data (including those of 127 brain tumor patients and 54 healthy people) were segmented using the FCM-based segmentation method. An image segmented by the improved method was randomly selected. The segmentation process is shown in Figure 3.

### 2.6. Statistical Analysis

All experimental data were statistically analyzed by SPSS 26.0, and measurement data were expressed as the mean + standard deviation (*x*(_) ± *s*), while enumeration data were statistically inferred by *χ*^2^ test. The measurement data conformed to normal distribution were tested using *t*-test, the rank sum test was performed for those did not conform to normal distribution, and a difference was considered statistically significant as *P* < 0.05. The receiver operator characteristic (ROC) curve was utilized for analyzing the sensitivity, specificity, and optimal diagnostic threshold of each index for grading diagnosis of glioma. The calibration level *α* = 0.05, *P* < 0.05, indicated the difference to be statistically significant.

## 3. Results

### 3.1. Verification Results of the Algorithm

**Figure 4:**
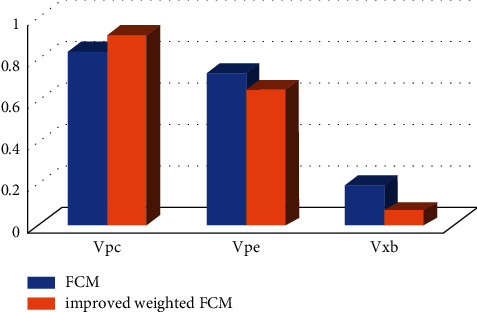
Performance comparison between the original algorithm and the improved FCM algorithm in image segmentation.

As shown in Figure 4, the improved FCM algorithm had the higher Vpc and lower Vpe compared with the original algorithm. This suggested that the membership matrix of the proposed improved algorithm had a lower degree of ambiguity and a higher segmentation accuracy. The results also showed that the Vxb was lower, indicating that the intraclass pixels were more closely related and the interclass pixels were more irrelevant. In general, the improved algorithm had obvious advantages in the process of image segmentation.

### 3.2. Experimental Results of Image Segmentation

**Figure 5:**
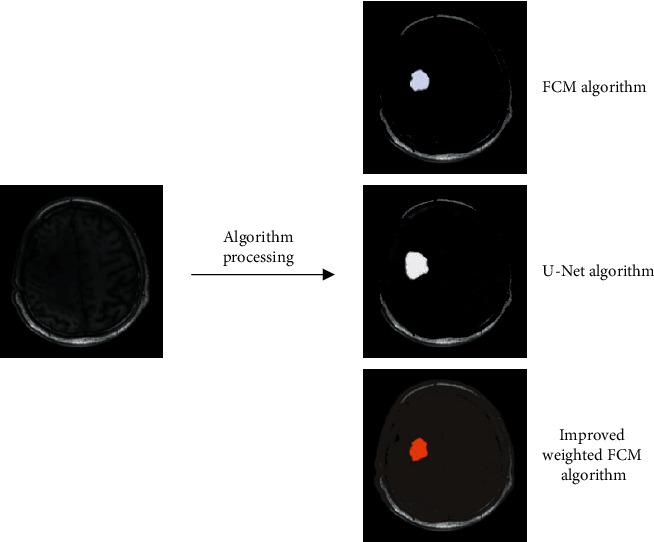
The brain tissue image segmented by the improved FCM method.

The original FCM algorithm, the U-Net algorithm, and the improved FCM algorithm were used for the processing of the brain tumor MRI images. As the segmentation results are presented in Figure 5, the segmentation effect of the improved FCM algorithm was better than other algorithms.

Usually, the segmentation result would be compared with the gold standard to complete the analysis of the segmentation. However, brain MRI images represented complex brain tissue structures, and the gold standard segmentation became time-consuming and labor-intensive, making it difficult to realize. Therefore, statistical methods were usually used for analysis, and the experimental results were evaluated and analyzed from an indirect perspective to verify the validity and accuracy of the segmentation. With the continuous development of computer technology, machine learning methods have been widely used in medical image analysis with their own advantages. Medical image processing methods could be better evaluated, and the accuracy of their analysis results could also be improved. Therefore, the FCM segmentation method and the improved FCM segmentation method were compared and analyzed from the aspects of volume calculation and machine learning classification. The segmented data were divided into groups of low-grade glioma, high-grade glioma, and healthy control objects for analysis and comparison, to verify the accuracy of the improved segmentation method.

**Figure 6:**
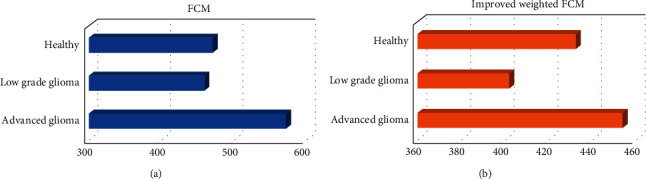
Comparison of gray matter volume in different groups. (a) The gray matter volume calculated by the FCM algorithm; (b) the gray matter volume calculated by the improved FCM algorithm.

Volume calculation has been often used in quantitative analysis methods. It was simple in operation and accurate in results and widely used in the analysis of medical images, especially brain MRI images. Here, the segmented and registered images were statistically organized in groups and categories, and the average volume of gray matter in each group was calculated. Compared with the healthy group, high-grade glioma showed the higher gray matter volume and white matter volume, while those of lower-grade glioma group were lower.  Figure 6 shows the gray matter volume in different groups.

**Figure 7:**
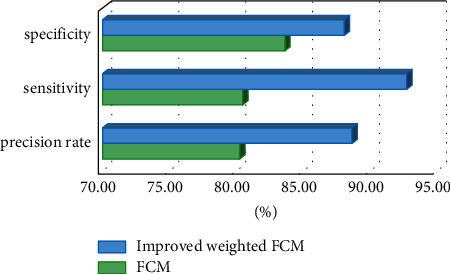
Comparison of specificity, sensitivity, and accuracy.

For the comparison of classification results, there have been more and more types of medical images, and related medical aided diagnosis technologies were also widely used. As medical images were closely combined with computer-aided diagnosis technology, the use of computer-aided diagnosis could improve the accuracy of diagnosis. It could also recognize and process various medical images and detect lesion areas. After feature extraction, the method of using pattern recognition and classification has become one of the important methods for diagnosis in medical images with the aid of computers. The comparison of classification results is shown in Figure 7.

Figure 7 shows that the improved FCM could give a higher accuracy in segmenting images, which reached 88.64%. It had a higher sensitivity to gray matter changes in brain tumors, reaching 92.72%. Its specificity was not much different from that of traditional FCM, which were 83.61% and 88.06%, respectively.

### 3.3. Analysis Results of MRS

**Figure 8:**
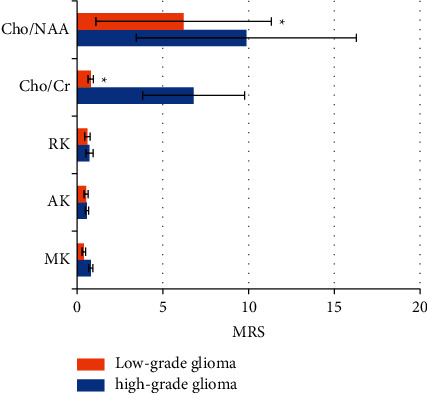
Comparison of parameters between high-grade gliomas and low-grade gliomas.

After statistical analysis, the mean kurtosis (MK), axial kurtosis (AK), and radial kurtosis (RK) values of high-grade gliomas and low-grade gliomas were of statistically significance. The differences in the ratio of choline/creatine (Cho/Cr) and the ratio of choline/N-acetyl-aspartate (Cho/NAA) were statistically significant. The results are shown in Figure 8.

The diagnostic value of the five parameters was compared, and the ROC curve was used as shown in Table 2. The area under the curve (AUC) of the MK was the largest, which was 0.962, *P* < 0.001, and the best critical value was 0.4096. It was followed by RK, AK, Cho/NAA, and Cho/Cr, whose AUC value was 0.834, 0.792, 0.841, and 0.803, respectively; *P* values were all less than 0.05, and the best critical values were 0.6435, 0.5641, 6.8752, and 2.6843, respectively.

## 4. Discussion

Brain tumor is one of the major diseases that threaten human health. In its clinical diagnosis, MRI is one of the most common imaging methods. The MRI images of brain tumors are three-dimensional, and different types of brain tumors showed different characteristics and presented different states. The manual segmentation of brain tumor images is a heavy and time-consuming task, so it is the current trend to study the brain tumor image segmentation [17]. The outcome of clustering coincides with the goal that people want to achieve using image segmentation, so clustering algorithms are widely used in image segmentation [18]. FCM algorithm is one of the classic algorithms; it utilizes fuzzy thinking to describe the ambiguity of the objective world. FCM algorithm is widely applied because of its simple process and easy implementation, but it still has defects in many aspects [19].

To get better segmentation effect of FCM, the FCM algorithm was improved in this work, as the two-dimensional hybrid algorithm was improved to a three-dimensional one. In this case, MRI images of brain tumors and MRS were taken for analysis, which were of 127 brain tumor patients in the low-grade glioma group as well as 54 healthy persons in the healthy group. The results show that the improved FCM algorithm had a higher Vpc (0.9154, 0.8352) and a lower Vpe (0.6516, 0.7326), and the cluster validity index Vxb (0.0621, 0.1931) was lower. Such a result was consistent with the views of Hua et al. [20]. The improved FCM algorithm had obvious advantages in the image segmentation process, which were mainly reflected in lower ambiguity of membership matrix, higher segmentation precision, and the correlation between intraclass pixels and interclass pixels.

The analysis of gray matter volume suggested that compared with the healthy group, the gray matter volume of the high-grade glioma was higher (453.87), and that of the low-grade glioma group was lower (401.99). The improved FCM algorithm had a high accuracy in segmenting images, which reached 88.64%. It also had a higher sensitivity to gray matter changes in brain tumors, reaching 92.72%. Its specificity was not much different from that of the original FCM, which were 83.61% and 88.06%, respectively. Halder and Talukdar [21] also mentioned in their article that the pattern recognition classification method at current was one of the important methods for the diagnosis of medical images with the aid of computers. The improved FCM algorithm had high accuracy and sensitivity, which allowed it better assist doctors in medical image recognition processing, lesion area detection, etc., improving the accuracy of doctors' clinical diagnosis. For studying the diagnostic value, the results demonstrated that the AUC of the MK value was the largest, which was 0.962; and the optimal critical value was 0.4096, which had a greater reference significance for clinical treatment and prognosis. Significant differences were also found in Cho/NAA and Cho/Cr between the high-grade and low-grade gliomas, but the specificity and sensitivity were slightly poorer. Therefore, a certain guiding significance could be offered for the grading of gliomas.

## 5. Conclusion

The improved FCM algorithm was applied to the MRI images of low-grade glioma patients as well as healthy people in this research, and the performance detection and diagnostic value of the improved FCM algorithm were studied. As a result, the improved FCM algorithm had a better image segmentation effect and the higher accuracy and sensitivity compared with the original FCM. It was helpful to clinicians in diagnosing brain tumors. The disadvantages of this work were that the details were not fully handled, and there was no complete data comparison. In the future, it was planned to improve the segmentation theory in this work, further promoting the accuracy of the segmentation outcomes. On the other hand, the glioma was simply classified into the high-grade and low-grade types merely for the limitation of experimental data. There was not a detailed classification according to the classification of World Health Organization and the specific types of gliomas. Thus, the classification would be studied in detail as a large number of cases would be collected subsequently. This work provided a theoretical reference for the computer-based clinical diagnosis of brain tumors.

## Figures and Tables

**Table 1:** Imaging parameters of MRI.

Items	Parameters	Functions
Time of repetition (*θa*)	1980 ms	It determined the value of *θ*1.
Delay time of echo (*θb*)	2.28 ms	The contrast of *θ*2 would be affected.
Frequency encoding direction	245 mm^*∗*^245 mm^*∗*^165 mm	The smaller the value, the higher the resolution.
Flip angle	8°	It defined echo pulse sequence.
Volume of a unit voxel in a brain image	1 mm³	

**Table 2:** Comparison of the diagnostic value of different parameters for high-grade and low-grade gliomas.

Parameters	ROC	Critical value	Sensitivity	Specificity	*P*
MK	0.962	0.4096	0.928	0.867	<0.001
AK	0.834	0.6435	0.742	0.546	0.024
RK	0.792	0.5641	0.646	0.642	0.007
Cho/Cr	0.841	6.8752	0.725	0.545	0.018
Cho/NAA	0.803	2.6843	0.762	0.531	0.009

## Data Availability

The data used to support the findings of this study are available from the corresponding author upon request.
